# Changes in MOTS-c Level in the Blood of Pregnant Women with Metabolic Disorders

**DOI:** 10.3390/biology10101032

**Published:** 2021-10-12

**Authors:** Małgorzata Wojciechowska, Ewa Pruszyńska-Oszmałek, Paweł A. Kołodziejski, Hanna Krauss, Natalia Leciejewska, Dawid Szczepankiewicz, Jakub Bień, Marek Skrzypski, Maciej Wilczak, Maciej Sassek

**Affiliations:** 1 Department of Mother and Child Health, Poznan University of Medical Sciences, ul. Polna 33, 60-535 Poznań, Poland; malgorzata59@onet.eu (M.W.); kzmid@gpsk.am.poznan.pl (M.W.); 2 Department of Animal Physiology, Biochemistry and Biostructure, Poznan University of Life Sciences, 60-637 Poznań, Poland; ewa.pruszynska@up.poznan.pl (E.P.-O.); pawel.kolodziejski@up.poznan.pl (P.A.K.); natalia.leciejewska@up.poznan.pl (N.L.); dawid.szczepankiewicz@up.poznan.pl (D.S.); jakub.bien@up.poznan.pl (J.B.); marek.skrzypski@up.poznan.pl (M.S.); 3 Department of Medicine, The President Stanislaw Wojciechowski State University of Applied Sciences in Kalisz, Nowy Świat 4, 62-800 Kalisz, Poland; hjk12@poczta.fm

**Keywords:** MOTS-c, obesity, mothers’ blood, umbilical cord blood

## Abstract

**Simple Summary:**

Metabolic relationships between mother and child are currently some of the most studied in the context of maternal imprinting. This is particularly important in the context of metabolic diseases as well as newly discovered peptides, proteins, and biologically active substances produced in the bodies of a mother and child. One of them is MOTS-c, which belongs to the group of mitochondria-derived peptides (MDP). The first reports show that it plays an important metabolic role in carbohydrate–lipid metabolism. We decided to investigate the concentration changes in MOTS-c levels in maternal and umbilical cord blood at delivery in healthy, obese, and hypothyroidism subjects. We found changes in MOTS-c levels depending on the metabolic condition of mothers.

**Abstract:**

MOTS-c peptide is a member of the group of mitochondria-derived peptides (MDP). It is a product of the open reading frame in the 12S RNA gene. Due to its features and functions in the body, this peptide is classified as a hormone. The first publications indicated that this hormone improves insulin sensitivity and lowers body weight in obese animals. This suggests that it may be an important peptide in maintaining the body’s energy homeostasis. The aim of our work was to investigate the potential role of MOTS-c peptide during pregnancy, which is a condition prone to metabolic disorders. The research covered healthy, obese women and women with thyroid disorders. The obtained results indicated an increase in the concentration of MOTS-c in the blood of mothers and newborns in the obese group as compared to the healthy control group and a corresponding decrease in the concentration of this peptide in mothers and newborns in the group with hypothyroidism compared to the obese group. Moreover, we also observed a strong positive correlation between the concentration of MOTS-c in maternal blood and in umbilical cord blood. In summary, the MOTS-c peptide shows changes in blood concentration in various physiological states and may, in the future, become an important tool in the fight against metabolic diseases such as obesity or type 2 diabetes.

## 1. Introduction

MOTS-c is one of the most intriguing peptides and belongs to the mitochondria-derived peptides (MDP) family. It was first discovered in 2015 by Lee et al. [1]. The term “MOTS-c” is an acronym defined as the *m*itochondrial *o*pen-reading-frame of the *t*welve *S* rRNA type-*c*. MOTS-c is the second member of the MDP family and was discovered almost 20 years ago.

The first member of the MDP family, humanin, was discovered in 2001 and 2003 by three independent teams using different methods [2,3,4]. The unique feature of MOTS-c and humanin peptides is their localization within the sequence coding for mitochondrial 12S RNA. Moreover, based on its systemic actions in organisms, the MOTS-c peptide is classified as a hormone [1] and can serve as a transcription factor [5]. MOTS-c is a product of an open reading frame that is a 51 bp segment, and the mature peptide has 16 aa. Since its discovery, a number of reports describing the influence of MOTS-c on the metabolism have been published. The significant role of MOTS-c was first documented in the works by Lee et al. [1,6,7]. The authors showed that MOTS-c is a crucial element in energy homeostasis and metabolic processes. It was shown to improve diet-induced obesity, decrease muscle insulin resistance, stimulate glucose uptake, and enhance pentose and purine synthesis [1,6] Moreover, it was shown that the MOTS-c protein level is regulated by androgens (DHT—dihydrotestosterone) in combination with insulin in gravid uterine and placental ferroptosis in PCOS-like rats [8]. Improvement of insulin sensitivity through MOTS-c has been shown to be closely related to the adiponectin pathway [9]. In addition, it has been shown that in pre-diabetic and diabetic individuals, blood levels of MOTS-c decrease significantly [10]. Moreover, MOTS-c can extend the lifespan of cells and possibly the whole organism [11,12]. It is interesting to note that MOTS-c is closely related to physical activity and exercise [13,14].

The importance of MOTS-c in metabolism is a compelling reason to analyze its role in pregnancy, which is associated with an increased risk of metabolic abnormalities [15,16,17]. Therefore, this study aimed to investigate the potential changes in the levels of MOTS-c in healthy pregnant women and women with metabolic disorders such as obesity and hypothyroidism, and to document the metabolic state of these women by blood biochemical analysis. The second aim of the study was to explore the possible production of MOTS-c in newborns and measure its levels in cord blood shortly after birth.

## 2. Materials and Methods

### 2.1. Ethics

The study was conducted according to the principles stated in the Declaration of Helsinki. The protocol was approved by the Clinical Research Ethics Committee of Poznan University of Medical Sciences (approval number 997/18).

### 2.2. Material

All subjects in this study were Caucasian. The test material used was maternal peripheral blood collected on the day of childbirth (MB) and umbilical cord blood (CB). The parameters recorded for mothers included in the study were as follows: age (years), height (cm), body weight (kg), body mass index (BMI, kg/m^2^), and body weight just after birth. Obesity was defined as a BMI value of >30 according to the WHO standards [18]. The parameters measured for newborns were as follows: body weight (kg), head circumference (cm), chest circumference (cm), abdominal circumference (cm), thigh circumference (cm), and arm circumference (cm). The data on the characteristics of mothers and newborns are presented in Table 1 and Table 2 [19].

### 2.3. Metabolic and Hormonal Profile in Blood Serum

Metabolic parameters were determined in the blood samples using commercially available colorimetric tests. The levels of glucose, cholesterol (total, high-density lipoprotein, and low-density lipoprotein), and triglycerides (TGs) were measured using kits from Pointe Scientific according to the manufacturer’s instructions. The sample and reagent were analyzed using 96-well microplates after their volume was adjusted using the micro method. Absorbances were read on a Synergy 2 apparatus (Biotek, Winooski, VT, USA). Hormone concentrations were determined by enzyme-linked immunosorbent assay (ELISA) or radioimmunoassay (RIA) kits—serum MOTS-c levels were determined using ELISA kits (test range 4.985–600 pg/mL, sensitivity: 2.659 pg/mL, cat. no: 201-11-3562, Sunred, Shanghai, China), while other hormones were measured by the following tests: insulin—Human Insulin-Specific RIA (test range: 2–200 µU/mL; cat. no: HI-14K, Merck Millipore, Burlington, MA, USA), adiponectin—Adiponectin ELISA (test range: 0.6–31,000 µg/L; cat. no: E09, Mediagnost, Reutlingen, Germany), and leptin—Multi-Species Leptin RIA (test range: 1–50 ng/mL; cat. no: XL-85K, Merck Millipore, Burlington, MA, USA). For RIA tests, gamma radiation was quantified using the Wallac Wizard 1470 Gamma Counter (Perkin Elmer, Waltham, MA, USA). ELISA measurements were carried out on a Synergy 2 microplate reader (Biotek, Winooski, VT, USA) [20,21].

### 2.4. Statistical Analysis

Statistical analysis was performed using unpaired Student’s t-test and Mann–Whitney test. All data were normally distributed except for the hypothyroidism groups, where the Mann–Whitney test was used. Statistical significance was assumed at *p* < 0.05 and *p* < 0.01 and denoted by * and **, respectively, in comparison to the control group. The # and ## symbols were used by analogy but in comparison to the obese group. On the column graph, the letters are indicators of statistical differences and *p* < 0.05 and *p* < 0.01 are denoted by lowercase and uppercase letters, respectively, in comparison to the columns indicated by the letters. Correlations between the levels of MOTS-c in maternal blood and umbilical cord blood were analyzed using Pearson’s correlation model and linear regression. All statistical analyses were conducted in GraphPad Prism 6.0 software (GraphPad Software, San Diego, CA, USA).

## 3. Results

### 3.1. Body Weight and General Metabolic Profile

The body weight recorded before pregnancy and BMI unequivocally indicated obesity in one group of analyzed women. On the other hand, female subjects with hypothyroidism showed a lower body weight as well as BMI in comparison to obese subjects. A similar trend of body weight and BMI was also observed in the analyzed women on the day of birth (Table 1). Several parameters measured in the newborns did not show any statistically significant differences (Table 2). A detailed description of the metabolic state of the women included in the study is presented in Table 3. Parameters such as TG and leptin concentrations were found to be significantly elevated, while the adiponectin level was diminished in the serum of the obese women group. These observations support the notion that this group of participants had obesity. Similar results were observed in women with hypothyroidism: a decrease in the levels of T3, fT3, and T4 and an increase in the level of leptin (Table 3).

### 3.2. MOTS-c Concentration Changes

**Figure 1:**
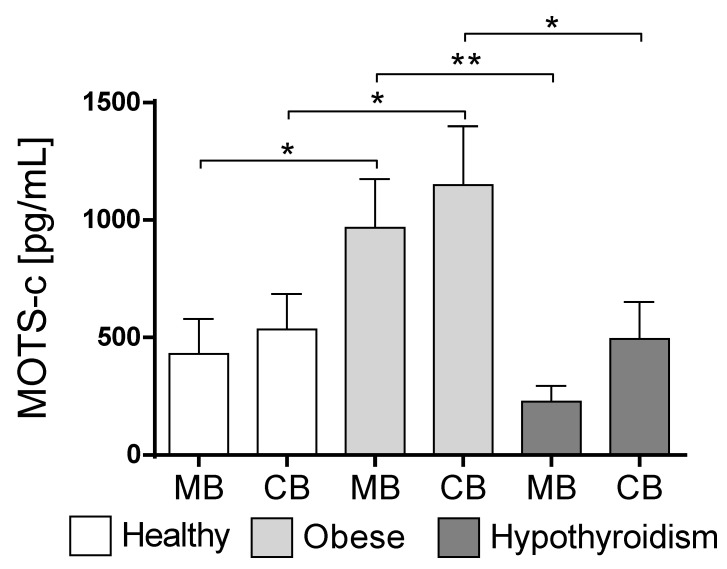
Changes in MOTS-c concentration in maternal and cord blood in non-obese and obese mothers. Values are presented as mean ± standard error of the mean (S.E.M.). Statistically significant differences are marked for *p* < 0.05 (*) and *p* < 0.01 (**). MB—mother’s blood; CB—cord blood.

No significant differences were found among the MOTS-c concentrations in the maternal blood serum and cord blood serum in the healthy group (Figure 1). Similarly, no differences were noticed in the obese group or in subjects with hypothyroidism. Interesting differences were noted between the MOTS-c levels in the maternal blood serum of the healthy women and obese women, and a significant increase in this level was found in obese women (*p* < 0.048, Figure 1). Similarly, an increased concentration of MOTS-c was observed in the cord blood in the obese group (*p* < 0.045, Figure 1).

A distinct decrease in MOTS-c levels was observed in maternal blood in the subjects with hypothyroidism in comparison to obese women (*p* < 0.003, Figure 1). Similarly, a decrease in MOTS-c concentration was observed in the cord blood in subjects with hypothyroidism compared to the obese group (*p* < 0.039, Figure 1).

**Figure 2:**
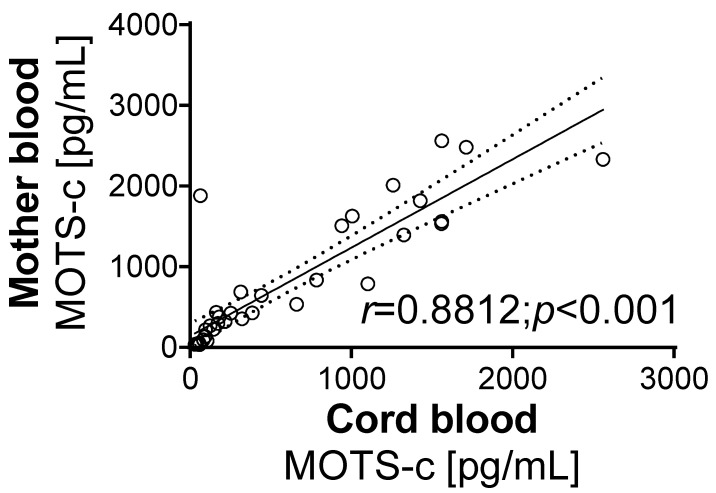
Correlation between MOTS-c level in mother’s blood and umbilical cord blood. Values for *r* and *p* are indicated in the graph. Solid and dashed lines show the means and 95% confidence intervals, respectively, following linear regression analysis. The *r*-value indicates correlation, and the *p*-value indicates the significance of the correlation.

The correlation analysis of the MOTS-c levels determined in all groups showed a strong positive correlation between the hormone levels in maternal blood and cord blood (*r* = 0.8812, *p* < 0.001, Figure 2).

## 4. Discussion

The obtained results shed some light on the role of MOTS-c in an organism. Until now, this peptide has received relatively little attention, and thus each piece of information could be important in the future. We provide novel findings derived directly from human physiology that add to our understanding of MOTS-c.

First, the biometric body features and biochemical characteristics of the participants were analyzed. The obtained data confirmed the physiological state of the healthy, obese, and hypothyroidism women included in the study. Similarly, biometric analysis was performed for newborns, which showed no significant differences. Furthermore, the metabolic and hormone profiles of all three groups of pregnant women with different metabolic states were explored. Similar parameters were measured in cord blood collected immediately after birth. The main part of the research analyzed the concentration of MOTS-c in maternal and cord blood and the correlation between the levels of MOTS-c in maternal blood and cord blood, which is a component of the newborn circulation.

The potential role of MOTS-c peptide in the regulation of metabolism and energy homeostasis seems to be of importance [1,22,23]. As stated above, MOTS-c is a product of the mitochondrial open reading frame of the 12S RNA gene and thus potentially present in all cells of an organism. The 12S gene is constantly transcribed, and the further formation of MOTS-c results from the subsequent translation mechanisms. This feature can be important for the metabolism of energy substrates and maintaining the energy balance in each cell. These assumptions seem to be true because MOTS-c has been reported to influence the metabolism of both carbohydrates and lipids [1,24,25]. Hence, we decided to investigate the levels of MOTS-c in pregnant women as pregnancy can be considered an important factor modifying the energy balance [26,27].

In our study, we did not observe any changes between maternal blood and cord blood in any of the analyzed groups. Moreover, we found a clear correlation between the MOTS-c levels in maternal blood and cord blood. This may suggest two facts: first, MOTS-c is expressed in the mother’s tissues and can easily cross the placental barrier; second, MOTS-c is also expressed in the placental tissue and in the fetus but cannot cross the placental barrier. Both assumptions can be true, but further analysis is needed to confirm one of them. Unfortunately, we did not have access to additional tissues (e.g., placental tissue) to at least partially verify these hypotheses, which is the main limitation of our work. The next step is to determine the expression of MOTS-c in the placenta as well as its immunofluorescent localization in the cells of this organ. This is important because MOTS-c has also been shown to regulate the metabolism of human placenta-derived mesenchymal cells [28].

Second, our results showed a clear increase in MOTS-c in obese mothers’ blood in comparison to healthy control women. This observation confirms that MOTS-c is an important factor regulating metabolism, possibly also as a factor in preventing damage to pancreatic islets [29]. An increase in the level of MOTS-c implies that the organism attempts to maintain high glucose utilization and limit the growth of adipose tissue. The increased concentration of MOTS-c in cord blood in the healthy and obese groups supports this assumption and allows it to be extended to the growing fetus as well. Interestingly, a similar increase was not observed in children, men, or non-pregnant women. This is probably due to a significant physiological difference in the state of pregnancy compared to other organisms [23,30].

Third, a decrease in the concentration of MOTS-c was observed in maternal blood and cord blood in subjects with hypothyroidism in comparison to the obese group. Hypothyroidism is generally characterized by a lower metabolism rate and possibly partially by a decrease in the MOTS-c level in the blood. We suppose that lower levels of MOTS-c can be one of the causes and an effect of hypothyroidism, which leads to a lower metabolism rate. In this context, MOTS-c can be a potential activator of metabolism, which could alleviate some of the symptoms of hypothyroidism.

## 5. Conclusions

In summary, MOTS-c, as a promising factor that plays a crucial role in the energy balance, should be intensively investigated. Understanding the role of this peptide could help to partially explain some of the intricacies of human metabolism and perhaps develop novel therapeutic methods [31].

## Figures and Tables

**Table 1:** Characteristics of mothers.

PARAMETER	Non-Obese (n-18)	Obese (n-22)	Hypothyroidism (n-17)
BEFORE PREGNANCY			
Women’s age (years)	30.09 ± 1.300	32.62 ± 1.243	29.75 ± 1.448
Height (cm)	168.3 ± 1.571	167.8 ± 1.245	166.8 ± 1.422
Body weight (kg)	63.88 ± 1.204	90.08 ± 3.536 **	67.25 ± 3.112 ^##^
BMI (kg/m^2^)	22.54 ± 0.194	32.29 ± 1.107 **	24.32 ± 1.282 ^##^
ON THE DAY OF BIRTH			
Body weight (kg)	79.58 ± 1.903	98.32 ± 3.116 **	80.58 ± 2.861 ^#^

Values are presented as mean ± S.E.M. Statistically significant differences are marked for *p* < 0.01 (**). The # and ## symbols were used by analogy but in comparison to the obese group.

**Table 2:** Anthropometric parameters of newborns.

PARAMETER	Non-Obese	Obese	Hypothyroidism
Child gender (M/F)	9/9	10/12	10/7
Body weight (kg)	3.555 ± 0.146	3.544 ± 0.177	3.447 ± 0.090
Head circumference (cm)	36.11 ± 0.5536	36.3 ± 0.5287	36.06 ± 0.3147
Chest circumference (cm)	34.11 ± 0.5710	34.25 ± 0.5519	34.06 ± 0.3028
Abdominal circumference (cm)	34.33 ± 0.8205	35.35 ± 0.5679	34.65 ± 0.3202
Thigh circumference (cm)	12.39 ± 0.2574	12.5 ± 0.2565	12.24 ± 0.2016
Arm circumference (cm)	10.17 ± 0.2021	10.22 ± 0.2365	10 ± 0.1485

Values are presented as mean ± S.E.M.

**Table 3:** Metabolic characteristics of the study subjects.

Parameter	Non-Obese		Obese		Hypothyroidism	
	MB	CB	MB	CB	MB	CB
Glucose (mg/dL)	96.74 ± 4.39	88.70 ± 4.06	103.7 ± 2.62	97.32 ± 3.67	97.93 ± 2.72	90.01 ± 3.97
NEFA (mmol/L)	0.651 ± 0.055	0.408 ± 0.042	0.893 ± 0.094	0.418 ± 0.045	0.749 ± 0.081	0.542 ± 0.070
Cholesterol (mg/dL)	197.7 ± 9.46	185.1 ± 23.80	244.7 ± 8.79	172.6 ± 7.15	216.9 ± 11.66	206.4 ± 16.22
Triglycerides (mg/dL)	233.9 ± 24.23	135.4 ± 20.40	339.3 ± 28.74 **	121.7 ± 22.65	267.1 ± 28.43	155.5 ± 24.43
Adiponectin (µg/mL]	9.95 ± 0.72	35.06 ± 2.62	7.84 ± 0.60 *	35.22 ± 2.93	8.36 ± 0.87	32.04 ± 2.75
Leptin (ng/mL)	8.09 ± 0.45	7.59 ± 0.59	9.78 ± 0.28 **	6.84 ± 0.71	9.43 ± 0.22 *	6.55 ± 0.87
Insulin (ng/mL)	11.42 ± 1.23	3.53 ± 0.69	16.71 ± 2.76	9.25 ± 4.33	10.88 ± 1.56	3.98 ± 1.03
TSH (mIU/L)	1.62 ± 0.18	8.95 ± 1.59	2.13 ± 0.47	9.75 ± 1.87	1.57 ± 0.17	11.85 ± 2.52
T3 (ng/mL)	1.48 ± 0.07	0.64 ± 0.07	1.44 ± 0.06	0.75 ± 0.15	1.15 ± 0.09 *^#^	0.67 ± 0.05
FT3 (pg/mL)	2.451 ± 0.14	1.211 ± 0.13	2.235 ± 0.15	1.268 ± 0.20	1.917 ± 0.15 *	1.235 ± 0.21
T4 (µg/dL)	8.76 ± 0.43	8.68 ± 0.26	8.58 ± 0.31	8.10 ± 0.28	8.22 ± 0.41	8.74 ± 0.25
FT4 (ng/mL)	1.13 ± 0.03	1.07 ± 0.03	1.12 ± 0.03	1.15 ± 0.02	1.05 ± 0.02 *	1.10 ± 0.02

Values are presented as mean ± S.E.M. Statistically significant differences are marked for *p* < 0.05 (*) and *p* < 0.01 (**) vs. non-obese or *p* < 0.05 (#) vs. obese. MB—mother’s blood; CB—cord blood.

## Data Availability

The data presented in this study are available on reasonable request from the corresponding author.

## References

[1] Lee C., Zeng J., Drew B.G., Sallam T., Martin- A., Wan J., Kim S., Mehta H., Hevener A.L., De R. (2015). The Mitochondrial-Derived Peptide MOTS-c Promotes Metabolic Homeostasis and Reduces Obesity and Insulin Resistance. Cell Metab..

[2] Hashimoto Y., Niikura T., Tajima H., Yasukawa T., Sudo H., Ito Y., Kita Y., Kawasumi M., Kouyama K., Doyu M. (2001). A Rescue Factor Abolishing Neuronal Cell Death by a Wide Spectrum of Familial Alzheimer’s Disease Genes and Aβ. Proc. Natl. Acad. Sci. USA.

[3] Guo B., Zhai D., Cabezas E., Welsh K., Nouraini S., Satterthwait A.C., Reed J.C. (2003). Humanin Peptide Suppresses Apoptosis by Interfering with Bax Activation. Nature.

[4] Ikonen M., Liu B., Hashimoto Y., Ma L., Lee K.W., Niikura T., Nishimoto I., Cohen P. (2003). Interaction between the Alzheimer’s Survival Peptide Humanin and Insulin-like Growth Factor-Binding Protein 3 Regulates Cell Survival and Apoptosis. Proc. Natl. Acad. Sci. USA.

[5] Kim K.H., Son J.M., Benayoun B.A., Lee C. (2019). The Mitochondrial-Encoded Peptide MOTS-c Translocates to the Nucleus to Regulate Nuclear Gene Expression in Response to Metabolic Stress. Physiol. Behav..

[6] Lee C., Kim K.H., Cohen P., Angeles L., States U. (2016). MOTS-c: A Novel Mitochondrial-Derived Peptide Regulating Muscle and Fat Metabolism. Free. Radic. Biol. Med..

[7] Goetzl E.J., Wolkowitz O.M., Srihari V.H., Reus V.I., Goetzl L., Kapogiannis D., Heninger G.R., Mellon S.H. (2021). Abnormal Levels of Mitochondrial Proteins in Plasma Neuronal Extracellular Vesicles in Major Depressive Disorder. Mol. Psychiatry.

[8] Zhang Y., Hu M., Jia W., Liu G., Zhang J., Wang B., Li J., Cui P., Li X., Lager S. (2020). Hyperandrogenism and Insulin Resistance Modulate Gravid Uterine and Placental Ferroptosis in PCOS-like Rats. J. Endocrinol..

[9] Guo Q., Chang B., Yu Q.-L., Xu S.-T., Yi X.-J., Cao S.-C. (2020). Adiponectin Treatment Improves Insulin Resistance in Mice by Regulating the Expression of the Mitochondrial-Derived Peptide MOTS-c and Its Response to Exercise via APPL1–SIRT1–PGC-1α. Diabetologia.

[10] Ramanjaneya M., Bettahi I., Jerobin J., Chandra P., Khalil C.A., Skarulis M., Atkin S.L., Abou-Samra A.B. (2019). Mitochondrial-Derived Peptides Are down Regulated in Diabetes Subjects. Front. Endocrinol. (Lausanne).

[11] Fuku N., Pareja-Galeano H., Zempo H., Alis R., Arai Y., Lucia A., Hirose N. (2015). The Mitochondrial-Derived Peptide MOTS-c: A Player in Exceptional Longevity?. Aging Cell.

[12] Kołodziejski P.A., Pruszyńska-Oszmałek E., Wojciechowicz T., Sassek M., Leciejewska N., Jasaszwili M., Billert M., Małek E., Szczepankiewicz D., Misiewicz-Mielnik M. (2021). The Role of Peptide Hormones Discovered in the 21st Century in the Regulation of Adipose Tissue Functions. Genes (Basel).

[13] Woodhead J.S.T., Merry T.L. (2021). Mitochondrial-Derived Peptides and Exercise. Biochim. Biophys. Acta-Gen. Subj..

[14] Yang B., Yu Q., Chang B., Guo Q., Xu S., Yi X., Cao S. (2021). MOTS-c Interacts Synergistically with Exercise Intervention to Regulate PGC-1α Expression, Attenuate Insulin Resistance and Enhance Glucose Metabolism in Mice via AMPK Signaling Pathway. Biochim. Biophys. Acta-Mol. Basis Dis..

[15] Kapur A., McIntyre H.D., Hod M. (2019). Type 2 Diabetes in Pregnancy. Endocrinol. Metab. Clin. N. Am..

[16] Dow M.L., Szymanski L.M. (2020). Effects of Overweight and Obesity in Pregnancy on Health of the Offspring. Endocrinol. Metab. Clin. N. Am..

[17] Casey B.M., Leveno K.J. (2006). Thyroid Disease in Pregnancy. Obstet Gynecol..

[18] Caballero B. (2019). Humans against Obesity: Who Will Win?. Adv. Nutr..

[19] Warchoł M., Wojciechowska M., Kupsz J., Sot-Szewczyk M.H., Michalak M., Kołodziejski P., Pruszyńska-Oszmałek E., Krauss H. (2018). Association of Cord Blood Ghrelin, Leptin and Insulin Concentrations in Term Newborns with Anthropometric Parameters at Birth. J. Pediatr. Endocrinol. Metab..

[20] Kołodziejski P.A., Pruszyńska-Oszmałek E., Korek E., Sassek M., Szczepankiewicz D., Kaczmarek P., Nogowski L., Maćkowiak P., Nowak K.W., Krauss H. (2018). Serum Levels of Spexin and Kisspeptin Negatively Correlate with Obesity and Insulin Resistance in Women. Physiol. Res..

[21] Baylan F.A., Karaküçük S. (2021). Maternal Plasma Elabela Levels in Intrauterine Growth Restriction. Cukurova Med. J..

[22] Zarse K., Ristow M. (2015). A Mitochondrially Encoded Hormone Ameliorates Obesity and Insulin Resistance. Cell Metab..

[23] Cataldo L.R., Fernández-Verdejo R., Santos J.L., Galgani J.E. (2018). Plasma MOTS-c Levels Are Associated with Insulin Sensitivity in Lean but Not in Obese Individuals. J. Investig. Med..

[24] Lu H., Wei M., Zhai Y., Li Q., Ye Z., Wang L., Luo W., Chen J., Lu Z. (2019). MOTS-c Peptide Regulates Adipose Homeostasis to Prevent Ovariectomy-Induced Metabolic Dysfunction. J. Mol. Med..

[25] Ramanjaneya M., Jerobin J., Bettahi I., Bensila M., Aye M., Siveen K.S., Sathyapalan T., Skarulis M., Abou-Samra A.B., Atkin S.L. (2019). Lipids and Insulin Regulate Mitochondrial-Derived Peptide (MOTS-c) in PCOS and Healthy Subjects. Clin. Endocrinol..

[26] Ladyman S.R., Augustine R.A., Grattan D.R. (2010). Hormone Interactions Regulating Energy Balance during Pregnancy. J. Neuroendocrinol..

[27] Most J., Broskey N.T., Altazan A.D., Beyl R.A., St. Amant M., Hsia D.S., Ravussin E., Redman L.M. (2019). Is Energy Balance in Pregnancy Involved in the Etiology of Gestational Diabetes in Women with Obesity?. Cell Metab..

[28] Yu W.D., Kim Y.J., Cho M.J., Seok J., Kim G.J., Lee C.H., Ko J.J., Kim Y.S., Lee J.H. (2021). The Mitochondrial-Derived Peptide MOTS-c Promotes Homeostasis in Aged Human Placenta-Derived Mesenchymal Stem Cells in Vitro. Mitochondrion.

[29] Kong B.S., Min S.H., Lee C., Cho Y.M. (2021). Mitochondrial-Encoded MOTS-c Prevents Pancreatic Islet Destruction in Autoimmune Diabetes. Cell Rep..

[30] Du C., Zhang C., Wu W., Liang Y., Wang A., Wu S., Zhao Y., Hou L., Ning Q., Luo X. (2018). Circulating MOTS-c Levels Are Decreased in Obese Male Children and Adolescents and Associated with Insulin Resistance. Pediatr. Diabetes.

[31] Dabravolski S.A., Nikiforov N.G., Starodubova A.V., Popkova T.V., Orekhov A.N. (2021). The Role of Mitochondria-Derived Peptides in Cardiovascular Diseases and Their Potential as Therapeutic Targets. Int. J. Mol. Sci..

